# “Defendit Numerus”: A Pooled Analysis of 6145 Locally Advanced Rectal Cancer Treated with Preoperative Chemoradiotherapy

**DOI:** 10.3390/jcm13185456

**Published:** 2024-09-14

**Authors:** Jacopo Giuliani, Marta Mandarà, Marco Muraro, Elvira Rampello, Antonella Franceschetto, Francesco Fiorica

**Affiliations:** Department of Oncology, Azienda ULSS 9 Scaligera, 37122 Legnago, VR, Italy; jacopo.giuliani@aulss9.veneto.it (J.G.); marta.mandara@aulss9.veneto.it (M.M.); marco.muraro@aulss9.veneto.it (M.M.); elvira.rampello@aulss9.veneto.it (E.R.); antonella.franceschetto@aulss9.veneto.it (A.F.)

**Keywords:** rectal cancer, radiochemotherapy, fluoruracil-based chemotherapy, control group

## Abstract

**Objective:** The optimal management of rectal cancer remains a subject of ongoing research. This meta-analysis of individual patient data assessed the benefit of chemoradiotherapy (fluorouracil-based) in local advanced rectal cancer: disease-free survival and overall survival. **Methods:** We pooled the data of 6145 patients from 24 studies of rectal cancer who received neoadjuvant radiotherapy with concomitant fluorouracil or capecitabine and surgery. The PRISMA 2020 abstract checklist was followed. Individual participant survival was reconstructed with an algorithm from published Kaplan–Meier curves. **Results:** The median OS was not reached; the mean survival time was 135.4 months (127.9–141.5). The median DFS was 176.9 months, and the mean disease-free survival time was 122.6 months (111.7–131.9). **Conclusions:** We provided a benchmark for future studies on rectal cancer treatment. The present results can be used in decision-making for locally advanced rectal cancer patients.

## 1. Introduction

Rectal cancer is a significant healthcare burden worldwide; approximately 800,000 new rectal cancer diagnoses are expected, and about half have locally advanced rectal cancer [1]. Traditionally, surgical resection has been the cornerstone of treatment for rectal cancer. However, the treatment paradigm has evolved in these years. In 2000, a meta-analysis [2] of clinical trials evaluating the efficacy of preoperative radiotherapy demonstrated promising results, leading to the widespread adoption of this approach as a standard treatment. Also, it has been proven that therapeutic effects can be ameliorated by associating chemotherapy with radiotherapy [3]. The goals of chemoradiotherapy in rectal cancer should increase the results obtained with radiotherapy (downstaging the tumour, the likelihood of sphincter preservation and local tumour control) and reduce the risk of distant metastasis and more impactful overall survival (OS) outcomes [4]. With the gold-standard treatment, less than 5% of patients will have a local recurrence disease, but a stable rate of patients will have distant metastasis [3]. Even today, the optimal management of rectal cancer remains a subject of ongoing research. Particularly, several attempts have been made to evaluate the impact of chemotherapy-only treatment for reducing side effects on DFS and OS. Also, recently, a randomised trial of neoadjuvant chemotherapy showed no inferiority in disease-free survival of preoperative chemotherapy to preoperative chemoradiotherapy [5].

Considering the landscape of systemic treatments in the coming years, an accurate estimate of DFS and OS rates in this group of patients is essential. These data can allow us to evaluate the natural history of rectal cancer treated with chemoradiotherapy (fluorouracil-based), assess the treatment’s effect size, and translate the results into clinical practice.

The treatment’s priority is to decrease the distant recurrence rate. It needs to modify the microenvironment where the tumour grows.

Also, radiation treatment should no longer be considered a locoregional therapy but a systemic treatment capable of interacting with the tumour microenvironment and the immunity system. 

Changing the natural history of rectal cancer is the only modus to increase survival.

The aim of this study was to assess the DFS and OS in patients with locally advanced rectal cancer treated with chemoradiotherapy using a fluorouracil-based regimen. Furthermore, we can make benchmarking a crucial factor in evaluating a new treatment modality’s efficacy and safety, determining the patient outcomes compared to standard chemoradiotherapy. The results of this study may guide future research efforts aimed at optimising treatment strategies and improving long-term survival outcomes in rectal cancer patients.

## 2. Methods

### 2.1. Structure of This Study

The present study combines individual patient data (MIPD) [6] of DFS and OS from randomised controlled trials (RCTs) in patients with locally advanced rectal cancer treated with chemoradiotherapy using a fluorouracil-based regimen.

### 2.2. Study Selection and Qualitative Analysis

A systematic search of MEDLINE was performed for articles published up to 31 December 2022 with no lower date limit, including the following keywords: “rectal cancer”, “preoperative radiochemotherapy” and “RCTs”.

Studies were included if they met all the following criteria:

(1) Data were in the English language and accessible in the form of full-text papers, presentations, posters or oral presentations;
(2) The target population of the original study was patients with locally advanced rectal cancer who were treated with preoperative chemoradiotherapy using a fluorouracil-based regimen;
(3) The study evaluated disease-free survival and overall survival outcomes after the treatment.

When the results of a single study were reported in more than one publication, only the most recent and complete data were included.

We assessed the quality of the trials according to each separate component, with a maximum possible score of ten points [7]. Studies that were published only in abstract form were excluded from the analysis. This decision was likely made because assessing methodological quality can be challenging when only abstracts are available. Full-text papers, posters or oral presentations typically provide more comprehensive information about study design, methods and results than abstracts.

### 2.3. Reconstruction of Survival Data

In clinical trials with a time-dependent outcome (death or disease progression), survival curves were used to describe the risk of the event over time. Individual participant data (IPD) were obtained by reconstructing survival data from the published Kaplan–Meier curves.

For reconstructing survival probabilities, the algorithm developed by Guyot et al. was used [8]. This algorithm provides accurate predictions for survival time and the expected event of interest (i.e., alive or dead; disease progression or no progression) and shows excellent accuracy for survival probabilities and medians. The algorithm uses he digitalised data on survival probabilities, time and total number of patients, and events to find numerical solutions to the inverted Kaplan–Meier equations. The data were digitalised using GetData Graph Digitizer (version 2.26.0.20), a free, open-source tool for extracting numeric data from images or graphs.

Each reconstructed survival curve, OS or TTP, was visually inspected for accuracy to the original Kaplan–Meier curve by overlapping the obtained curve with the published one and comparing the median OS/TTP published with those reconstructed. We used the nonparametric approach reported by Combescure et al. [9] to assess pooled survival probabilities from several single-arm studies. This approach is a version for aggregated data of the product-limit estimator of survival and uses random effects to model between-study heterogeneity.

The between-study covariance matrix was estimated using the multivariate extension of DerSimonian and Laird’s method [10,11]. Compared to meta-analyses of survival probabilities at a single time point [12], this approach has several advantages. First, the estimation of the pooled survival probability at time *t* also involves all studies ending before *t* because these studies contribute to the estimated conditional survival probabilities for time intervals prior to *t*. Second, this approach does not require assumptions about the shape of survival curves. Finally, the pooled survival probabilities are guaranteed not to increase over time. For all analyses, a *p*-value < 0.05 was considered statistically significant. All analyses and graphics were completed with the R Statistical Computing Environment (R Foundation for Statistical Computing, Vienna, Austria). 

The primary outcomes were overall survival rate (ORR) and disease-free survival (DFS).

Two expert reviewers independently assessed selected studies in the systematic search (JG and MM). Any discrepancies were resolved by discussion with a third expert reviewer (FF).

## 3. Results

### 3.1. Baseline Characteristics

The pooled study cohort consisted of 6145 individual rectal cancer patients fulfilling eligibility criteria and receiving preoperative fluorouracil-based radiochemotherapy. These patient data were extrapolated from 24 studies. All of these studies are randomised clinical trials.

Methodological quality scores varied from 7 to 10 on a scale of 2 to 10. All the included trials reported an adequate efficacy of randomisation and an adequate follow-up.

A high-quality score (≥7 points) was observed in all trials. Inclusion criteria were uniform in all but one RCT, which included only unresectable patients [13].

The main features of included patients are summarised in Table 1. The median patient age was 68.5 years with a range from 54 [14,15] to 64.5 years [16]. The number of males was 4141 (68.5%). Almost all patients had good performance: 2644 (71.2%) patients had a WHO 0, while 820 (20.1%) patients had a WHO 1.

The proportion of patients with stage T3 was 66.9%, ranging from 18.75% [13] to 100% [17]. The percentage of N-positive patients was 56.9%, ranging from 37.9% [17] to 91% [18]. The proportion of patients with tumours between 4 and 8 cm was 44.5%, ranging from 31.8% [19] to 62% [20]; those with tumours less than 4 cm was 40.1%; and the remaining 13.5% had tumours localised above 8 cm.

**Table 1:** Study- and patient-level characteristics of the studies in the meta-analysis.

Authors/Trial	N°	Median Age (Years)	Male	ECOG PS			Anal Verge (cm)				pT Stage				pN Stage		
				0	1	2	<4	4–8	>8	NA	pT1-2	pT3	pT4	NA	pN0	pN1-2	NA
Aschele et al. [21] **STAR-01**	379	63	259	330	248	1	89	202	81	7	7	307	65	0	134	242	2
Appelt et al. [18]	111	62	68	NA	NA	NA	NA	NA	NA	NA	0	90	21	0	10	101	0
Bahadoer et al. [22] **RAPIDO**	450	62	312	NA	NA	NA	115	153	151	31	14	299	137	0	35	415	0
Bosset et al. [23] **EORTC 22921**	253	62	183	202	50	0	125	114	14	0	0	230	23	0	22	208	0
Bujko et al. [13]	48	59	33	22	24	2	24	0	0	24	0	96	36	0	NA	NA	NA
Conroy et al. [24] **UNICANCER-PRODIGE 23**	230	62	156	182	44	0	83	118	29	0	2	188	35	0	22	208	NA
Deng et al. [14] **FOWARC**	165	54	103	NA	NA	NA	90	70	5	0	8	100	57	0	37	128	0
Gérard et al. [25] **ACCORD 12/0405-Prodige 2**	293	63	191	229	43	1	204	0	89	0	23	255	15	0	85	205	3
Hofheinz et al. [16]	392	65	260	216	138	4	NA	NA	NA	NA	65	290	29	0	147	232	0
Jiao et al. [26]	103	63	68	82	16	5	25	57	21	0	3	61	39	0	23	80	0
Jin et al. [27] **STELLAR**	297	56	208	254	43	0	148	149	0	NA	9	250	38	NA	49	248	NA
Jung et al. [20]	71	56	44	45	26	0	17	44	10	0	0	57	14	0	8	63	0
Kairevice et al. [28]	72	63	49	NA	NA	NA	30	37	5	0	4	52	16	0	21	51	0
Rouanet et al. [29] **GRECCAR-1**	265	63	169	NA	NA	NA	NA	NA	NA	NA	20	217	18	0	105	160	0
Allegra et al. [30] **NSABP04**	641	58	433	NA	NA	NA	NA	NA	NA	NA	NA	NA	NA	NA	NA	NA	NA
Mohiuddin et al. [31] **RTOG**	50	56	38	35	15	0	NA	NA	NA	NA	0	34	16	0	31	19	0
Appelt et al. [17] **TTROG**	161	64	120	87	71	3	31	88	42	0	0	161	0	0	90	61	10
Park et al. [15]	107	54	67	NA	NA	NA	64	43	0	0	1	105	1	0	35	72	0
Rödel et al. [32] **CAO/ARO/AIO-04**	623	63	440	475	141	0	216	336	64	7	32	537	50	4	159	451	0
Sauer et al. [33] **CAO/ARO/AIO-94**	406	62	293	NA	NA	NA	NA	117	189	85	15	NA	NA	NA	NA	NA	NA
Schmoll et al. [34] **PETACC 6**	547	62	394	420	126	1	236	311	0	0	39	466	42	0	118	393	25
Salazar et al. [35]	100	60	65	65	35	0	35	41	22	0	1	83	15	0	11	89	0
Valentini et al. [19] **INTERACT**	280	60	188	NA	NA	NA	188	89	3	0	28	250	0	2	58	220	2
Total	6145	62	4141	2644	820	17	1837	2041	621	84	256	4114	667	44	1218	3499	42

Legend: N = number of patients, ECOG PS = Eastern Cooperative Oncology Group Performance Status, NA = not available.

### 3.2. Overall Survival

**Figure 1:**
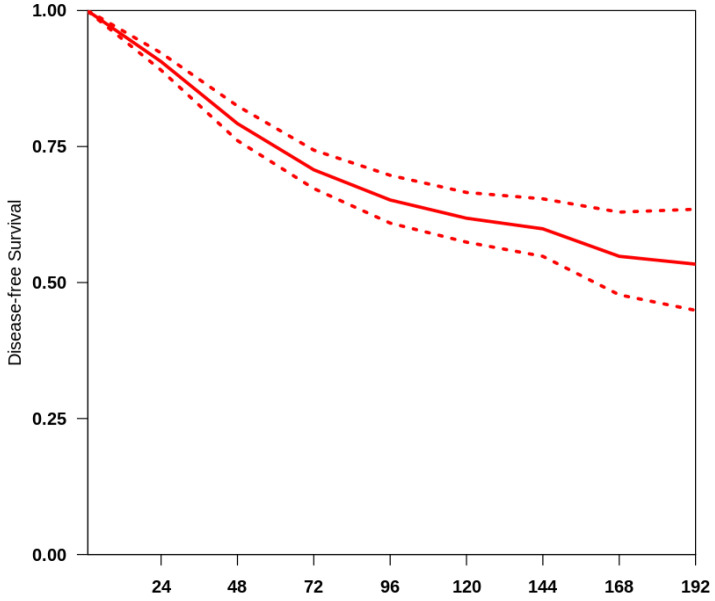
Curve of OS in the studies included. The thick red line represents the summarised overall survival curves with the 95% confidence bands (dashed lines) obtained using the approach of Combescure et al. [9] with random effects.

The overall median follow-up of all the studies included was 48 months. We obtained a summary survival curve from the survival probabilities and numbers of at-risk patients collected at various time points (Figure 1). The 3-, 5- and 10-year OS probabilities with their 95% CI were 70.9% (66.2–75.9%), 65.8% (61.3–70.7%) and 62.5% (58.2–67.2%), respectively. The median survival time was not reached; the mean survival time was 135.4 months (127.9–141.5). There was evidence of only moderate between-study heterogeneity (I^2^ = 64.8%).

### 3.3. Disease-Free Survival

**Figure 2:**
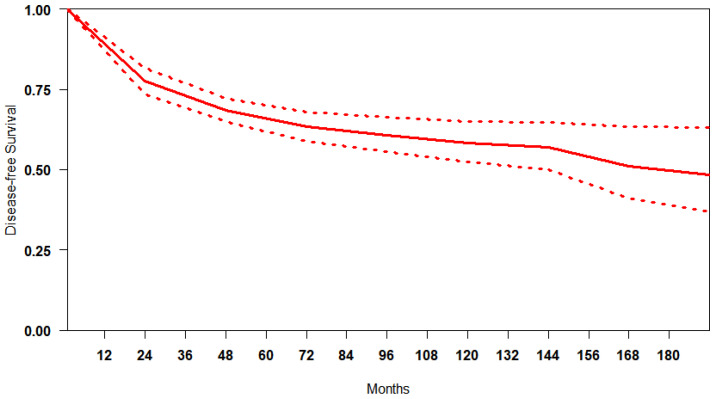
Curve of DFS in studies included. The thick red line represents the summarised DFS curves with the 95% confidence bands (dashed lines) obtained using the approach of Combescure et al. [9] with random effects.

Reconstruction of IP data for DFS was feasible for 5764 patients. We obtained a summary DFS curve from the DFS probabilities and numbers of at-risk patients collected at various time points (Figure 2). The 3-, 5- and 10-year disease-free survival probabilities with their 95% CI were 70.9% (66.2–75.9%), 65.8% (61.3–70.7%) and 60.9% (58.7–63.2%), respectively. The median survival time was 176.9 months; the mean survival time was 122.6 months (111.7–131.9). There was evidence of only moderate between-study heterogeneity (I^2^ = 74.6%).

## 4. Discussion

Preoperative chemoradiotherapy, using fluorouracil-based regimens, has become the gold standard for treating locally advanced rectal cancer. This approach has significantly improved oncological outcomes and substantiated hope of controlling the disease. The principal hypothesis behind the combined approach is to induce tumour downsizing and hopefully downstaging, increase the likelihood of a conservative surgical resection and contemporaneously increase the possibility of local control. 

By shrinking the tumour before surgery, chemoradiotherapy can reduce the tumour’s involvement with critical structures, allowing surgeons to perform less extensive resections that preserve organ function and maintain the patient’s quality of life. This approach is particularly important in cases where the tumour is close to the anal sphincter, as it can potentially avoid the need for a permanent colostomy.

Perhaps exceeding all expectations, the published randomised clinical trials demonstrated significant improvements in outcomes that extended well beyond this primary objective. Some of these trials revealed that patients treated with the combined preoperative approach experienced remarkable long-term survival rates, with median disease-free survival reaching unpredictable durations and overall survival curves not yet reaching their median even after more than a decade. These outcomes suggest that this treatment approach, even if acting locally, carried out its function systematically, reducing distant metastases and enhancing overall survival. 

In oncology, it is rare to observe a survival curve extended to a median DFS of 176 months and a median OS not reached even after 15 years. Something which was achieved in locally advanced rectal cancer using the combination of preoperative radiation therapy, fluorouracil-based chemotherapy and subsequent surgery.

The relative awareness of this achievable good prognosis has brought about concerns about overtreatment in these patients which could lead to unnecessary costs, potential side effects and reduced quality of life. Establishing a clear benchmark for rectal cancer treatment is essential.

Data from various clinical trials can play a pivotal role in creating these benchmarks. By systematically analysing and comparing outcomes from different studies; clinicians can develop evidence-based standards that guide treatment decisions. This process can not only help to integrate effective new therapies in clinical practice but also prevent the premature elimination of effective treatments that are beneficial, ensuring that patients receive the best possible care based on comprehensive evidence

In rectal cancer, the belief that radiotherapy acts locally and the contemporary improvement in surgical techniques have caused some confusion regarding the real addictive value of combined treatment. The thought-stable rate of distant metastases in rectal cancer (often studied with colon cancer, partially ignoring the differences between the two) has highlighted a need for an improvement in the efficacy of systemic therapy, sometimes in spite of local control. Several attempts have been made to try to avoid radiation therapy to minimise the potential long-term side effects of radiation, such as bowel dysfunction, sexual dysfunction and secondary cancers. A Chinese RCT [14] failed to achieve its goals because the chemotherapy-alone approach did not improve systemic disease control and, more concerningly, worsened local disease control. A non-inferiority trial [5] concluded that preoperative chemotherapy could serve as an initial treatment for rectal cancer, reserving radiation therapy for specific cases. The trial design allowed radiation therapy to be omitted in patients who responded well to chemotherapy and did not show signs of progression. These and other studies underscore that the omission of radiation therapy in rectal cancer is not suitable for all patients. A selective approach was proposed, where radiation is possible but only for patients who do not achieve a good response with chemotherapy or who have specific risk factors (mesorectal fascia invasion or lateral lymph node metastasis after treatment). This chance involves refining diagnostic tools and biomarkers to assess the patient’s response to chemotherapy and identifying clearly the specific risk factors. We must remember that omitting RT reduces, surely, the local control and, potentially, the overall survival. It is essential to remember that a non-inferiority clinical trial intrinsically admits a clinically acceptable efficacy loss, 5% of 5-year disease-free survival [5]. Defining “acceptable efficacy loss” is critical for the sample size calculation and depends on clinical judgment (subjective for definition) and knowledge of the effect of standard chemoradiotherapy.

Considering only morphological and geographical tumour information, the decision to omit radiotherapy in favour of systemic chemotherapy for patients with rectal cancer, particularly those with distal tumours requiring abdominoperineal resection, tumours invading adjacent organs or tumours with predicted circumferential resection margin (CRM) involvement, is complex and should be made on a case-by-case basis. It is essential to emphasise that the decision to omit radiotherapy in favour of systemic chemotherapy should be made in consultation with a team of experienced healthcare professionals specialising in rectal cancer treatment.

They will carefully weigh the risks of local recurrence against the potential benefits and side effects of radiation therapy and chemotherapy. The goal is to provide each patient with the most effective and personalised treatment plan while minimising unnecessary interventions and associated risks. Knowing the natural history of rectal cancer treated with a preoperative approach is essential.

Our study comprehensively analysed combined survival data from individual preoperative groups in twenty-four phase III randomised controlled trials.

This method overcomes the limitations associated with using aggregate data, thereby increasing the precision of the statistical analysis and refining the accuracy of effect size estimates.

The goal of this study, which utilised individual patient data (MIPD), was to evaluate the DFS and OS rates in patients with locally advanced rectal cancer following neoadjuvant chemoradiotherapy (fluorouracil-based). 

This analysis can identify the non-inferiority margin for other clinical trial designs in rectal cancer and provide a benchmark for the published results. 

These data showed that individual patients’ median time for disease progression was 177 months. To our knowledge, the present study represents the largest (>6000 individuals) and up-to-date effort in conducting such an analysis in this clinical context. 

The generated actuarial survival curve for overall survival can serve as a valuable reference for determining the sample size needed for future trials of first-line new systemic treatment and for making indirect comparisons between different trials to estimate efficacy.

Likewise, the actuarial DFS curve is a robust estimate for assessing the risk of disease progression, offering a valuable benchmark for treatment decisions in locally advanced rectal cancer.

The methodology of this study may be influenced by the potential limitation of generalising its findings to other populations and settings, a common issue in meta-analyses. Although the studies included were conducted in highly specialised centres, the large sample size of over 6000 patients provides a solid basis for the conclusions. However, variability in baseline illness severity (different stages) and adjuvant chemotherapy protocols could affect the precision of our findings. We incorporated patient- and study-level covariates to control for these differences, but our analysis is limited by inconsistencies in the patient-level data reported across studies. As a result, it is important to note that these summary results reflect heterogeneity between studies rather than between individual patients, representing group averages rather than true individual effects. We could not account for other potentially important confounders (such as microscopic vascular invasion, histological grading and gene profiling) due to a lack of available data, which might have impacted our results. Despite the absence of individual-level data on patient covariates in this meta-analysis, we could still assess progression and survival flexibly.

The evidence from this meta-analysis of aggregate data, which allows for the assessment of outcomes as time-dependent events, is sufficient for concluding that in patients with locally advanced rectal cancer treated with preoperative fluorouracil-based chemoradiotherapy, the median DFS is 176.98 months. In contrast, the median OS has not yet been reached. These pooled actuarial OS and DFS probabilities offer a valuable benchmark for indirect comparisons.

## Data Availability

Dataset available on request from the authors.
